# Small Network for Lightweight Task in Computer Vision: A Pruning Method Based on Feature Representation

**DOI:** 10.1155/2021/5531023

**Published:** 2021-04-17

**Authors:** Yisu Ge, Shufang Lu, Fei Gao

**Affiliations:** College of Computer Science and Technology, Zhejiang University of Technology, Hangzhou 310023, China

## Abstract

Many current convolutional neural networks are hard to meet the practical application requirement because of the enormous network parameters. For accelerating the inference speed of networks, more and more attention has been paid to network compression. Network pruning is one of the most efficient and simplest ways to compress and speed up the networks. In this paper, a pruning algorithm for the lightweight task is proposed, and a pruning strategy based on feature representation is investigated. Different from other pruning approaches, the proposed strategy is guided by the practical task and eliminates the irrelevant filters in the network. After pruning, the network is compacted to a smaller size and is easy to recover accuracy with fine-tuning. The performance of the proposed pruning algorithm is validated on the acknowledged image datasets, and the experimental results prove that the proposed algorithm is more suitable to prune the irrelevant filters for the fine-tuning dataset.

## 1. Introduction

In the last decades, the rapid development of deep learning is promoted, and various novel neural networks are emerging endlessly, especially the convolutional neural networks (CNNs). In image classification, the convolutional layer was playing a more and more important role. However, with the improvement of the CNNs performance, the number of network parameters increased as well, which makes the CNNs difficult to apply in practical application. Moreover, there is a large number of redundant parameters in the CNNs structure [1], which influences the speed of network propagation seriously. Therefore, researchers were paying more and more attention to network compression in recent years and put forward various solutions for this problem. The compression methods in CNNs can be mainly classified into four categories: structure optimization, quantization and precision reduction, knowledge distillation, and network pruning. From RCNN [2] and FastRCNN [3] to FasterRCNN [4], the network structures were modified to accelerate the inference speed by reducing the number of repeated calculations. Similarly, MobileNet [5] was advised by Google in 2017, which deduces the number of parameters by the factorization of convolutional layers. After that, Sandler et al. [6] provided MobileNetV2 to amend the network by adding the residual structure. Except for the structure optimization on RCNN and MobileNet, the serial of IGCV [7–9] improved the network performance though interleaving group convolutions. Although the above structure optimization methods had great success, it is hard to design a new structure to improve network efficiency. Therefore, quantization and precision reduction were studied for equipping the CNNs in embedded equipment. Choi et al. [10] measured the weights of the network parameters through the Hessian matrix and quantified the network based on minimum weight quantization error. Zhou et al. [11] optimized the network in training by adding the sparse constraint into the loss function and compressing the sparse matrix in convolutional layers. Peng et al. [12] compressed the network via filter group approximation. Courbariaux et al. [13] introduced a method to train Binarized-Neural Networks. Rastegariy [14] put forward the XONR-Net to further optimize the binarized network by converting the input contents to the binarized type as well. The binarization helps the forward propagation be faster, but it depends on specific hardware, and the precision of networks cannot meet the sophisticated requirements. Not only were network structure modifications promoted, but also the training ways were paid much more attention. In 2015 NIPS, Hinton et al. [15] proposed the knowledge distillation to train small networks by big one, which showed the great potential of teacher-student mode and was the outset of the knowledge distillation. Since then, knowledge distillation has attracted widespread attention of scholars as a new direction of network compression, and methods [16–18] push the knowledge distillation to a more useful and explanatory stage. But knowledge distillation also faced the problems of difficult convergence and poor interpretability in training. Different from the above works, network pruning is much simpler and more useful. To find a suitable network structure for the fine-tuning dataset, a feature representation-based pruning algorithm is proposed, and the main contributions of this paper are presented as follows:

1. Samples and filters are linked in the proposed algorithm through feature representation, applied to find the irrelevant filters in the pretrained network.
2. Different from other pruning algorithms, the proposed algorithm aims to find the suitable network structure for practical tasks, and the pruned filters are redundant for the task on fine-tuning dataset.
3. The proposed algorithm is verified on multiple datasets and pretrained networks. The acknowledged datasets are applied to prove the effectiveness of the proposed idea, and the experimental results are encouraging and interesting.

The rest of this paper is structured as follows: Section 2 introduces the related works about the network pruning methods; the proposed pruning algorithm based on feature representations is described in Section 3; in Section 4, the proposed method is generalized to the multiple samples pruning; Section 5 gives the experiments, and the conclusion is provided in Section 6. The introduction should be succinct, with no subheadings. Limited figures may be included only if they are truly introductory and contain no new results.

## 2. Related Works

Network pruning algorithm has been widespread concerned, which is one of the most immediate and effective ways to compact and accelerate the CNNs. The most notable one must be the “Deep Compression” proposed by Han et al. [19] in 2016 ICLR. By eliminating the lightweight parameters, fine-tuning, weight sharing, and Huffman coding, the network is compressed above ten times to be applied in the embedded devices. The “Deep compression” shows great power in network compression, but the propagation speed of the pruned network is traded off because of the weight sharing approach. Li et al. [20] utilized the L1 constraint to regulate the pruning weight, and the pruning filters were ranked by the absolute value of filters. The experiment proved that the big value filter plays a more critical role than the small one by comparing multiple pruning mechanisms. He et al. [21] proposed an iterative two-step algorithm for pruning by a LASSO regression-based channel selection and least square reconstruction. Yu et al. [22] pruned network based on the neuron importance score propagation. Similarly, Molchanov et al. [23] estimated the neuron contribution to the final loss and iteratively removed the node with smaller scores. Anwar et al. [24] introduced structured sparsity at various scales for CNNs and addressed the importance of decision problem using a particle filtering approach. To deal with facial recognition more quickly, sparse ConvNet was advised by Sun et al. [25]. In contrast to the above methods, Srinivas and Babu [26] combined similar convolutions to speed up the network propagation and deleted the filters whose weights are zero. With the same idea, Ding et al. [27] put forward an optimization approach named C-SGD, which trained several filters to collapse into a single point in the parameter hyperspace and then removed the identical filters. A layer-wise pruning method was proposed by Chen and Zhao [28], which investigated the features learned in the convolutional layers and pruned at a layer level. Interestingly, iterative pruning was used to add multiple tasks to a single network by Mallya and Lazebnik [29]. IKP pruning scheme was advanced by Yang et al. [30] for removing redundant weights at a fine-grained level and showed good performance in hardware accelerator. To prune the deep models for object detection, Ghosh et al. [31] analyzed the pruning approach about the detection networks and utilized the pruning technique based on agglomerative clustering for the feature extractor and mutual information for the detector.

Most of the pruning methods measured the weight of convolutions first, then pruned the lightweight filters, and fine-tuned the remaining network. Li et al. [20] compared several different pruning mechanisms and proved that most of the big weight filters are more important in the network. Nevertheless, some layers are sensitive, in which pruning the lightweight filters will affect the accuracy of the entire network. Hence, some lightweight filters are pretty crucial as well, and the evaluation by weight of filters is not precise enough.

In most practical applications, the networks need only do a single job which is much easier than the object classification on ImageNet. Using the pretrained network may waste many computation resources. Therefore, the purpose of the pruning approach in this paper is to find an appropriate network for simple tasks. Considering the correlation between the source image and convolution feature maps, the CNNs pruning algorithm based on the feature representations is proposed, which bridges the relationships between the sample and convolutions, and guides the network to choose the desired filters.

The major advantages of the proposed method are listed as follows:

  Simple and useful: in the network propagation process, the convolutional layers are the most time-consuming part. Therefore, pruning the filters in convolutional layers is the most efficient way to accelerate the network. Irrelevant convolution will increase the operating burden of the system, and an effective pruning algorithm can make the network work more efficiently in practical applications. Comparing with other compression methods, network pruning is much simpler and easier to use.
  Easier fine-tuning: training from scratch in the same network structure may achieve the same performance [32], but the precondition is the powerful training equipment and large-scale training data. However, it is difficult to train small architectures from scratch [33] with limited training conditions. Fine-tuning from effective initial parameters makes the fine-tuning processing converge faster.
  More interpretable: the artificial neural network is a great technique, which is a black box and hard to be interpreted. Up to now, the complete explanation of the CNNs has not been given by any researcher. The proposed pruning method focuses on practical tasks and is guided by the correlation between the sample and feature representation. Compared with other pruning algorithms, the proposed method has better explanatory and more convincing.

## 3. Feature Representation-Based Pruning

In the forward propagation of simple CNNs, image data are fed into the network and processed layer by layer. Except for the final layer, each layer accepts the output of the former layer and outputs the intermediate results as the latter layer's input. As the output of convolutional layers, the feature representation implies the response of convolution filters to the input data. Hence, the feature representation links the filters with the input data, and the correlation between filters and samples can be revealed based on the feature representation. High correlation filters play an important in sample feature extraction, which can clearly distinguish objects from the background. Conversely, pruning the low correlation filters will not affect the network performance in practical application.

Therefore, how to use the feature representation to guide the filter selection is the major problem in this paper. Each convolution filter generates a feature map, and the feature map presents the response of the filter to the image. In general, a feature map is a gray image, and the brighter the feature map is, the stronger the response of the feature is. Therefore, the importance of the filter can be sorted simply by the brightness of the feature map. However, some feature maps with lower brightness might be important as well because the weight of the filter is smaller. So the feature maps need to be normalized, and the foreground and background are applied to evaluate the intensity of response in convolution filters.

**Figure 1:**
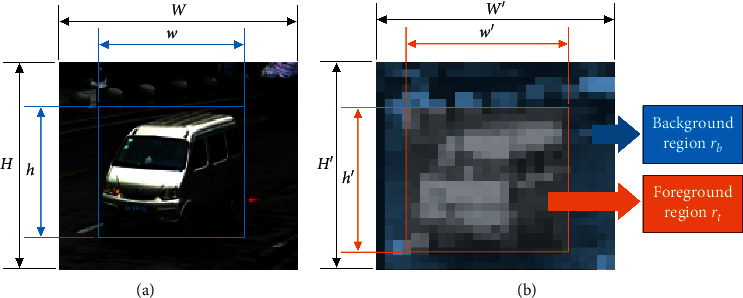
Illustration of feature response calculation. (a) Original image with label. (b) Feature map with label.

Training classification network only needs the class label while training detection network not only needs the labels of categories but also needs the locations of objects. In training, the detector will select the background area as the negative samples, and there is no need to prepare negative samples deliberately. Similarly, the proposed pruning algorithm needs the object location as well, and negative samples in training are also selected in the background. If the response intensity in the foreground region is stronger than that in the background region, the filter should be pruned. If the response intensity in the foreground region is stronger than that in the background region, the filter can be considered to extract the effective feature of the object; conversely, the redundant filter is hard to classify the object with background and should be eliminated by the pruning method. Suppose the pretrained network be *P*, which includes *n* convolutional layers. Also, suppose the pruning sample be *S* = (*I*, *label*), *label* = (*cx*, *cy*, *w*, *h*), where (*I*, *label*) is the sample image with the label, (*cx*, *cy*) is the center point of object location, and *w* and *h* mean the width and height of object bounding box. After forward propagation, feature maps *fm* in each convolutional layer are got, and irrelevant filters can be found based on feature representation. Before pruning the filters, the feature response of foreground within the bounding box *r*_t_ and the feature response of background *r*_b_ should be calculated at first according to equations (1)–(4), and the illustration is presented in Figure 1.

(1) $$\begin{array}{c} r_{t}=\underset{i}{\sum }\underset{j}{\sum }v_{ij\;},\\ \text{st}.x^{\prime }\leq i\leq x^{\prime }+w^{\prime },\\ y^{\prime }\leq j\leq y^{\prime }+h^{\prime },\\ v_{ij}\in fm, \end{array}$$

(2) $$\begin{array}{c} r_{b}=\underset{m}{\sum }\underset{n}{\sum }v_{mn}-r_{t},\\ \text{st}.0\leq m\leq W^{\prime },\\ 0\leq n\leq H^{\prime },\\ v_{mn}\in fm, \end{array}$$

(3) $$\begin{array}{c} \left\{\begin{array}{c} w^{\prime }=\frac{W^{\prime }\cdot w}{W}+\lambda_{l},\\ \begin{array}{c} h^{\prime }=\frac{H^{\prime }\cdot h}{H}+\lambda_{l},\\ \begin{array}{c} x^{\prime }=\frac{cx\cdot W^{\prime }}{W}-\frac{w^{\prime }}{2},\\ y^{\prime }=\frac{cy\cdot H^{\prime }}{H}-\frac{h^{\prime }}{2}, \end{array} \end{array} \end{array}\right) \end{array}$$

(4) $$\begin{array}{c} \lambda_{l}=\lambda_{l-1}+s_{l}-1, \end{array}$$

where (*x*', *y*') represents the left-top point of bounding box in feature map *fm*; *w*′ and *h*' mean the width and height of bounding box in *fm*, respectively; *W*′ and *H*′ are the width and height of *fm*; *W* and *H* denote the width and height of input image, respectively; *λ*_*l*_ is diffusion coefficients in *l*-th convolutional layer, which is used to evaluate the expansion of receptive field in feature map, *λ*_0_ = 0. Considering the effect of the edge features, the target area in the feature map is slightly larger than the original labeled position. Because of the expansion of the receptive field after each convolutional layer, the range of the foreground feature should be enlarged with the deepening of the network. However, considering the depth of the current network, the foreground feature range will exceed the scope of the feature map after several enlargements. Therefore, should the enlargement of the receptive field be considered? If yes, does it have any impacts on network pruning? Those questions will be discussed in the experimental part.

Since some objects in the background have similar features to the foreground objects, the feature response of the background and foreground will be similar. When the foreground region is small, only comparing with *r*_*t*_ and *r*_*b*_ will lead to wrong pruning. Hence, after calculating *r*_*t*_ and *r*_*b*_, it is necessary to compute the correlation *R* between the feature map *fm* and the object in the input images by the following equation:

(5) $$\begin{array}{c} R=\frac{r_{t}}{w^{\prime }\cdot h^{\prime }}-\frac{r_{b}}{W^{\prime }\cdot H^{\prime }-w^{\prime }\cdot h^{\prime }}. \end{array}$$

If *R* > 0, the feature map is relevant to the input image; conversely, the filter should be subtracted from the network. The same as other pruning methods, after pruning the former convolutional layer, the channel number and the corresponding weights in the latter layers need to be rectified.

**Figure 2:**
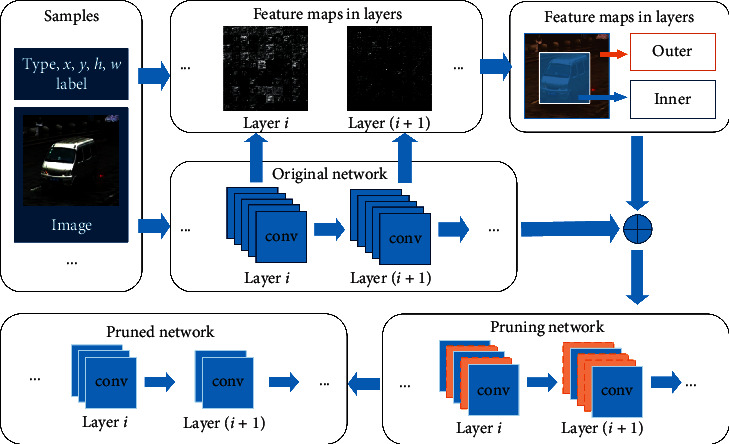
Flowchart of feature map-based pruning method.

After the pruning, the network needs to be fine-tuned for recovering the best performance. Each step is illustrated in Figure 2.

## 4. Pruning in Multiple Samples

If the above pruning method is used for network pruning iteratively, the more different samples are inputted into the pruning structure, the more filters are pruned. However, pruning based on different samples iteratively may affect the network feature extraction, which is misled by low relevance samples. To apply the pruning method in multiple samples, the pruning cartogram is advised in this section.

The same object in different images with identical viewing angle has similar representation features. For example, in the classification of people and vehicles, there are some differences between people individuals, but the essential people characteristics distinguish people from vehicles. Since people have legs and arms while vehicles have not, legs and arms are the essential features of people, directly classifying people and vehicles. Transforming to the pruning mechanism, although there are some differences between individuals, all the objects in the same category have identical features that are the essential features of this category. On the contrary, among different kinds of objects, different features are the main points to distinguish different objects, which need to be preserved. Thus, in multiple sample pruning tasks, the pruning cartogram is provided to count the pruning times of each filter. Similar to the pruning method based on filter weight, the pruning strategy in this part also depends on the pruning priority obtained by the pruning cartogram. Pruning times determine the correlation between the feature map and the object in the input images, and irrelevant filters are more frequently pruned. Suppose the pruning dataset be *D* = {(*I*_*k*_, *label*_*k*_) |*k* = 1, 2, 3, ...,*n*_*d*_}, where (*I*_*k*_, *label*_*k*_) is the *k*-th image with the label in the dataset and *n*_*d*_ is the number of samples in the dataset. Initialize the pruning cartogram *C*_o_ = {(*l*_*i*_, *f*_*ij*_, *c*_*ij*_)|*i* = 1, 2, 3, ..., *n*, *j* = 1, 2, 3, ...,*m*_*i*_}, where (*l*_*i*_, *f*_*ij*_, *c*_*ij*_) means that the *j*-th filter *f*_*ij*_ in *i*-th layer *l*_*i*_ has been pruned *c*_*ij*_ times and *c*_*ij*_ = 0; *m*_*i*_ denotes the filter number in *i*-th layer.

The correlation computation is the same as that in the single sample pruning method, which needs to calculate the foreground response *r*_t_ within the bounding box and the background response *r*_b_ out of bounding box at first and then get the correlation value *R* of the filter *f*_*ij*_ based on equation (5). If *R* < 0, add the 1 to *c*_*ij*_. After all the samples are computed, all the filters in the pruning cartogram are sorted in descending order of *c*_*ij*_, and the ranked pruning cartogram *C*_*r*_ = {(*l*_*i*_, *f*_*ij*_, *c*_*ij*_, *o*_*ij*_), *i* = 1, 2, 3, ..., *n*, *j* = 1, 2, 3, ...,*m*_*i*_} is obtained, where *o*_*ij*_ denotes the ranking result based on *c*_*ij*_. Finally, prune the filters which are satisfying equation (6), and fine-tune the pruned network.

(6) $$\begin{array}{c} o_{ij}<m_{i}\cdot \beta \wedge c_{ij}\neq 0. \end{array}$$

*β* means the pruning ratio, which is chosen by the users and will be verified in experiments.

Through the pruning algorithm mentioned above, the redundant filters are removed in the network based on the pruning cartogram. In the proposed pruning mechanism, the filters whose pruning count is equal to 0 will not be pruned, and the low relevance filters will be pruned preferentially. In the next section, adequate experiments are elaborated to measure the performance of the proposed method.

## 5. Results and Discussion

**Figure 3:**
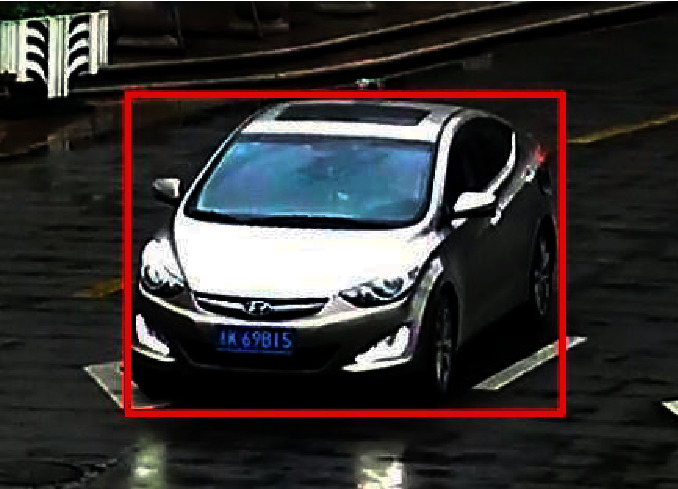
Sample in single image pruning.

**Figure 4:**
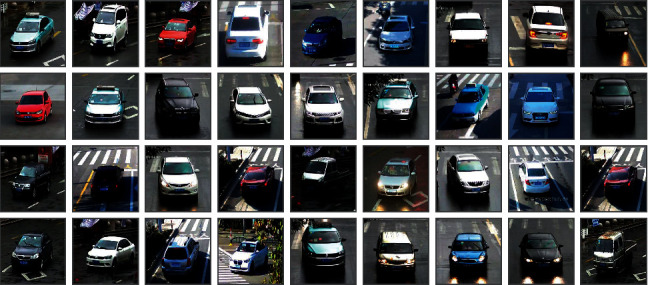
Pruning samples in multiple samples pruning.

Three CNN structures, that is, Cifar10-full [34], VGG16 [35], and YOLOV3 [36], are pruned in the experiments. The abovementioned CNNs have fewer parameters in full connect layers and are easy to be applied with the limited training resources. Datasets Cifar10 [37], ImageNet [38], and Pascal VOC [39] are applied, and the experiment is conducted on a computer with an Intel Core i7-8700k and an NVidia 1080 8G GPU. Cifar10-full is implemented on Caffe [34] on Windows10, VGG16 is implemented on both Caffe and Pytorch, and darknet [36] is utilized on Windows10 for YOLOV3.  Figure 3 shows the sample of single sample pruning, and the pruning samples in multiple sample pruning are shown in Figure 4. All the foreground objects are vehicles, and the background of all samples is street. In the experiment, Map-EX denotes the pruned model based on feature representation considering the receptive field expansion, which is proposed in the paper. Size-EX and scratch-EX are the networks with the same structure as Map-EX, but the initial fine-tuning parameters are different. Size-EX selects the filters based on the weight size of filters, and scratch-EX is trained from scratch. Map-NEX, Size-NEX, and scratch-NEX are the models without considering the receptive field expansion in network pruning, which means that *λ*_*l*_ in equation (4) is always 0. Size-EX and Size-NEX models measure the relative importance of filters in each layer by calculating the sum of its absolute weights and ranking by size, proposed by Li et al. [20], and will be called weight size pruning in the rest of the paper. Scratch-EX and scratch-NEX are the networks trained from scratch, which use the idea presented by Liu et al. [32]. For easy understanding, the proposed methods are called feature map pruning in the following sections. The details of the experiments are listed in Table 1.

### 5.1. Cifar10-Full on Cifar10

Cifar10-full is a lightweight network designed for the Cifar10 classification task consisting of 3 convolutional layers, 3 pooling layers, 2 local response normalization layers, and a full connect layer. The network is much easier in training than other complex and gigantic networks due to the small input size and lightweight network structure. Such a tiny network can more clearly reflect the impact of the pruning approaches and need not consider the overfitting problem. Cifar10 is a small image dataset, of which the image size is 32 × 32, and the classification number is 10. Each category has 60000 images, 50000 for training, and 10000 for testing. The categories include airplane, automobile, bird, cat, deer, dog, frog, horse, ship, and truck. To get the baseline accuracy for the network, Cifar10-full is trained from scratch.

The way of training Cifar10-full is similar to the method in [20], which trains 160 epochs for Cifar10 with a fixed learning rate 0.001. The constant learning rate and the fine-tuning time are also used to ensure enough training in the experiments. To verify the effectiveness of the proposed method, the experiments are designed as follow.

#### 5.1.1. Single Sample Pruning with Receptive Field Expansion

The receptive field will be enlarged with the propagation going deeper. In network pruning, whether the receptive field extension needs to be considered is answered as follows. Thus, the experiments show the influence of receptive field expansion as follows.

**Figure 5:**
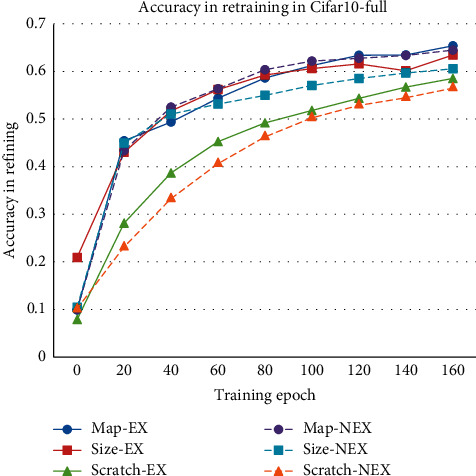
Single sample pruning with receptive field expansion.

As shown in Figure 5, the models considering the receptive field expansion have the same network structure and different initial weights, which compresses 30% of the network size. The models without considering the receptive field expansion have better performances in compression, which are compressed almost 63%, and the 1.3% accuracy decay is endurable. Whether considering the receptive field expansion or not, the convergence of the model based on the proposed method is better than others. Although the model based on weight size pruning has higher accuracy initially, model based on feature map pruning is potential. The model trained from scratch is hard to get the same result as other pruning methods got.

#### 5.1.2. Single Sample Pruning with Reduction of Classification Tasks

Only partial classes are required in the practical application, and the tasks are much easier than the challenge of ImageNet classification. Therefore, the pruned models are tested on a reduction of classification experiment, which imitates the practical situation for proving the effectiveness of the proposed method. The original model has trained 160 epochs in Cifar10 with the 0.001 learning rate, but the fine-tuning relies on the modified-Cifar10 that contains two categories: automobile and others. The automobile training part includes 5000 samples from Cifar10, and the other training part homogeneous selects 7000 samples from Cifar10 except for automobiles. The fine-tuning mechanism is the same as the original model training.

**Figure 6:**
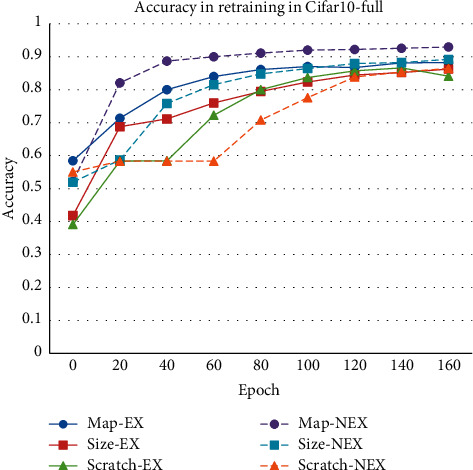
Single sample pruning with reduction of classification tasks.

As shown in Figure 6, the model based on feature map pruning still gets the best performance, which not only converges fastest but also gets the best performance after fine-tuning. Due to the classification reduction, all the models reach a higher accuracy than fine-tuning in the original Cifar10. In this training process, the model based on feature map pruning without considering the receptive field expansion obtains the best property, and unexpectedly, at the beginning of the fine-tuning, the model based on feature map pruning is better than that based on weight size pruning.

#### 5.1.3. Single Sample Pruning with Limited Training Samples

**Figure 7:**
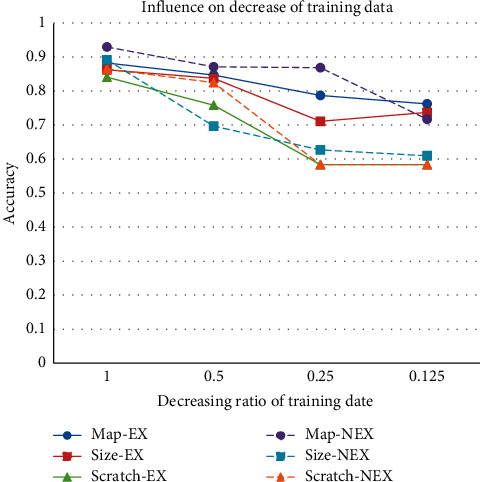
Single sample pruning with limited training samples.

Limited training data are the main reason why the pretrained model is necessary, and the training data are also one of the main factors that affect the model performance. To offer another confirmation of the proposed idea, an experiment with limited training samples is provided. In the experiment, the better-pruned network is less affected by the reduction of fine-tuning samples. The initialization parameters of the network are better, and the corresponding pruning algorithm is more appropriate. To evaluate the influence of limited training samples, the Cifar10 will keep being modified. In this part of the experiments, four different datasets are trimmed from modified-Cifar10. As shown in Figure 7, when the decreasing ratio is 1, it means that the fine-tuning dataset is the same as the modified-Cifar10 in the 2nd experiment, which only has two categories. The decreasing ratios, 0.5, 0.25, and 0.125, represent the samples in datasets that are selected from the modified-Cifar10, and the sample number of the datasets is half, quarter, and eighth of modified-Cifar10, respectively.

Curve trend in Figure 7 shows that the model performances deteriorate with decreasing the training data. The models based on the proposed method are still the best in the experiments. The model based on feature map pruning without considering the receptive field expansion performs great in sufficient training data, but the accuracy reduces a lot in eighth of training data. If the receptive field expansion is considered, the performance of the models is more stable in less training data.

#### 5.1.4. Multiple Samples Pruning with Different Pruning Ratios

In the practical application, the network performance and efficiency should be traded off, and to answer how to choose the right pruning ratio *β*, an experiment in multiple samples pruning with different pruning ratios is designed. In multiple samples pruning, 5000 vehicle samples are selected as pruning samples, strictly labeled by bounding boxes and similar to images in Cifar10, as shown in Figure 4.

In this part, the pruning ratios of 0.5, 0.6, 0.7, 0.8, and 0.9 are chosen. Literally, the 0.5 pruning ratio means that half of the filters in convolutional layers are removed, and the network size will be compressed to about a quarter of the original one in the full convolutional network. On account of the parameters in the other layers, network size after pruning is bigger than a quarter of the original one, and the size and accuracy of each pruned network are listed in Table 2.

In Table 2, the size of EX and accuracy of EX mean the size and accuracy of models considering the receptive field expansion; conversely, the size of NEX and accuracy of NEX mean the size and accuracy of models without considering the receptive field expansion. The accuracy of EX and accuracy of NEX are similar to the size of EX and size of NEX and no more introduction. Table 2 demonstrates the model based on the proposed method without considering the receptive field expansion. And the model whose pruning ratio is 0.8 gets the best trade-off between pruning size and network accuracy. Therefore, the receptive field expansion is superfluous. The small model not only occupies less computational resources but also is much easier to fine-tune.

**Figure 8:**
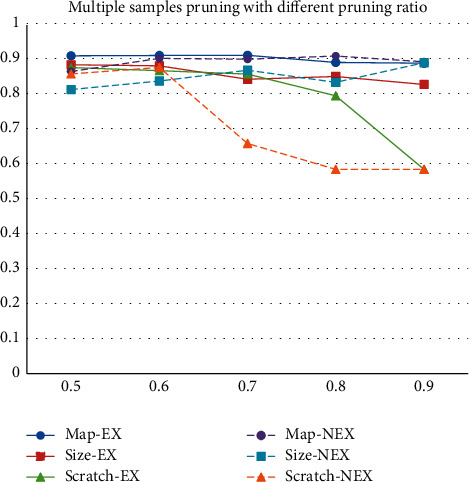
Multiple samples pruning with different pruning ratio.

**Figure 9:**
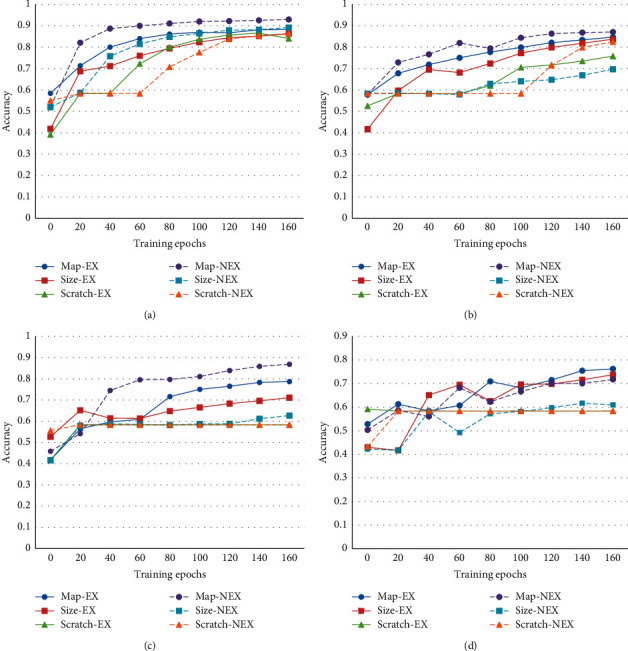
Fine-tuning processing in limited training data. (a) Refining in full data. (b) Refining in half of data. (c) Refining in quarter of data. (d) Refining in eighth of data.

The fine-tuning graph of multiple samples pruning with different pruning ratios is presented in Figure 8. Each network with the same pruning ratio has a uniform network structure but various initial weights. The fine-tuning process of limited training data is detailedly depicted in Figure 9, and the convergence curve becomes more and more winding with the decreasing of the training data. Models based on feature map pruning are more stable than other approaches. The performance of the model trained from scratch is decreasing along with the rise of the pruning ratio. The model based on pruning is more stable, and the feature map pruning-based models are still better than the weight size pruning-based ones. Although the weight size pruning-based model shows the potential in 0.9 pruning ratio, the proposed method-based model wins the competition by a narrow margin.

### 5.2. VGG16 on Cifar10 and ImageNet Subdataset

To further prove the performance of the proposed method and verify the pruning method used practically, the experiment of VGG16 on Cifar10 and ImageNet subdataset is constructed. Because the fine-tuning dataset is different from the pretraining dataset in practical, in the experiment, the pretraining dataset is Cifar10, and the fine-tuning datasets are the Cifar10 and the subdataset of ImageNet. VGG16 is a small network in VGG network serials, containing 13 convolutional layers and 3 full-connected layers. It is often equipped as the backbone of the detection network because of the great generalization performance. Therefore, it is popular in the field of network pruning. ImageNet is a large visual dataset containing 1000 categories, and each class includes 1300 samples for training and 50 for the test. It is one of the most famous datasets and has been widely used in artificial intelligence. To get the baseline accuracy for the experiment, VGG16 is trained from scratch to get the pretrained network. Following the work of Li et al. [20], VGG16 was pruned by the proposed method and the approach in [20], and the result is presented in Table 3. Meanwhile, multisample pruning (0.4) in the network is pruning by the proposed method with multiple samples, and the pruning ratio is 0.4, which means that 40% of filters on the network are pruned.

As shown in Table 3, the proposed method got a performance similar to what Li et al. [20] did. The comparison cannot show the progress of our algorithm because comparing with other networks on the original network deviates from the main point in this paper. The proposed pruning method tried to prune the redundant parameters when the complexity of the practical classification task is simpler than that in the pretraining dataset; particularly, the fine-tuning dataset is different from the training dataset. The model pruned based on the single sample feature map pruning gets the best performance in the experiment.

Therefore, a subdataset of ImageNet is utilized to prove the above idea. For further proving the effectiveness of the proposed method, network slimming algorithm [40] is implemented on VGG16, which is pretrained on Cifar10 and fine-tuned on the subdataset of ImageNet. The training batch size is 32, and the networks are trained in 160 epochs. The best model in training is kept for network performance evaluation. According to the choice of pruning sample in Figure 3, the taxi and street signs are selected as the subdataset of ImageNet, which means that the network should distinguish the taxi and street signs after fine-tuning. The result is listed in Table 4. The pruning ratio of each method is 0.6.

There is no doubt that network slimming is a great algorithm for network pruning, especially in the network compression on Cifar10, of which the precision is raised after the pruning and fine-tuning. But pruning for a practical application, the proposed method is more accurate than network slimming while network size after feature map pruning is smaller than that of network slimming. The network cannot be compressed in network slimming further because the bigger pruning ratio will eliminate all the filters in some layers.

### 5.3. YoloV3 on VOC

The proposed pruning method can be utilized not only in classification networks but also in the detection method with residual structure. The well-known network YOLOV3 is pruned in the experiment. YOLOV3 is a deeper network for object detection and is popular in academic and industry because of the real-time efficiency and great performance. Owing to the residual backbone of YOLOV3, it can prove that the proposed pruning method can also be used for residual network pruning. Pascal VOC dataset is a 20 class visual dataset, with 9963 images with 24640 annotated objects in VOC2007 and with 11530 images containing 27450 ROI annotated objects and 6929 segmentations in VOC2012. The data in VOC2007 and VOC2012 were used in the experiment for evaluating the performance in object detection. It is known that YOLO series networks are hard to train and adjust superparameters; therefore, the experiment on YOLOV3 is based on the model pretrained on ImageNet. The fine-tuning process is composed of two steps, and each step needs 80 epochs with a constant learning rate. In the first step of training, 0.001 is chosen as the learning rate and drops to one-tenth of its original level in step two. The evaluation is based on the mAP (mean Average Precision).

**Figure 10:**
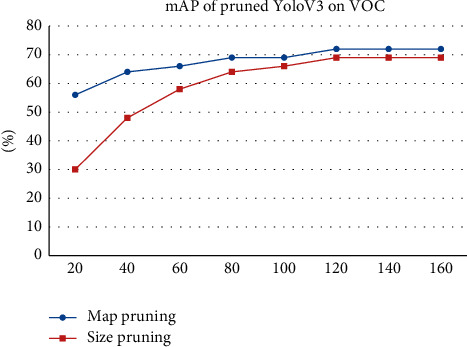
Fine-tuning process in YoloV3.

The results are shown in Figure 10, where map pruning denotes the model based on the feature map pruning way and size pruning means the model based on the weight size pruning mechanism, which is based on the algorithm proposed by Li et al. [20]. Although the two networks have the same size and structure, the initial parameters of fine-tuning are totally different because the pruning approaches choose different filters. In this experiment, the result proves that different initial parameters are important and pruning based on the feature map is better than using filter weights.

## 6. Conclusion

Focusing on the redundant parameters and hard training problems of neural networks, a convolutional neural network pruning algorithm based on feature representations is proposed. The feature maps of the convolutions in each layer are calculated through the network iteration. The response intensity of the foreground and background features is obtained according to the feature map with the bounding box label. Then the correlation between the filters and the object is bridged owing to the feature representations, which is the basis of the pruning algorithm. Further, the pruning strategy is extended from the single pruning sample to multiple pruning images. By extracting the essential features of the object, the pruning cartogram is advised to guide the pruning direction. The pruning operation is carried out based on the final pruning cartogram, which speeds up the network operation and is easy to fine-tune.

The proposed pruning algorithm is also verified on well-known datasets by some interesting experiments. The experimental results show that the proposed algorithm is better than the filter weight-based pruning method and training from scratch if the computation resources or training data are limited. For imitating the practical situation, the proposed method was also compared with another famous pruning algorithm in the experiment. The result presented that our pruning method is more suitable for pruning the redundant filters when the fine-tuning dataset is different from the pretraining dataset. And what is the optimal network structure? How to find that? How to train the network with little samples? Those are part of our ongoing work.

## Figures and Tables

**Table 1:** Detail of experiments.

No.	Original network	Pretraining	Refining	Superparameter	Pruning method
1	Cifar10_full	Cifar10	Cifar10	160 epoch 0.001lr	Map-EX, Size-EX, scratch-EX, Map-NEX, Size-NEX, and scratch-NEX
			Modified Cifar10		
			Modified Cifar10 with different data number		
			Cifar10	160 epoch 0.001lr	Multisample pruning with different ratio

2	VGG16	Cifar10	Cifar10	160 epoch 0.001lr	Map-NEX, multisample pruning (0.8), and network slimming
			ImageNet subdataset		

3	YoloV3	ImageNet	VOC	80 epoch 0.001lr; 80 epoch 0.001lr	Map-NEX; Size-NEX

**Table 2:** Size and accuracy of pruned network based on proposed method.

Pruning ratio	0.5	0.6	0.7	0.8	0.9
Size of EX (KB)	171	143	117	92	69
Accuracy of EX	90.8%	90.9%	90.9%	88.9%	88.7%
Size of NEX (KB)	101	71	49	**33**	19
Accuracy of NEX	86.3%	90.0%	89.8%	**90.7%**	89.1%

**Table 3:** VGG16 on Cifar10.

	Network size (M)	Pruned (%)	Test accuracy (%)
Li et al. [31]	21.1	64.0	90.51
Single sample pruning	16.4	72.0	**90.89**
Multisample pruning (0.4)	21.7	63.0	90.66
Multisample pruning (0.5)	15.1	74.2	89.98
Multisample pruning (0.6)	9.8	83.3	89.70

**Table 4:** VGG16 on subdataset of ImageNet.

	Network size (M)	Pruned (%)	Test accuracy (%)
Network slimming (0.6)	19.2	64.4	91.0
Single sample pruning	**14.2**	**73.7**	**94.0**
Multisample pruning (0.6)	25.2	53.3	91.0

## Data Availability

All data included in this study are available upon request by contact with the corresponding author.
